# The Role of the Reflective Thinking Scale for International Students in China Through Factor Analysis

**DOI:** 10.3390/bs15050651

**Published:** 2025-05-11

**Authors:** Jiangtao Fu, Ali Usman Hali

**Affiliations:** School of Foreign Languages, Henan University, Minglun Road, Kaifeng 450046, China; fujiangtao@henu.edu.cn

**Keywords:** reflective thinking scale, personal growth, factor analysis, self-awareness

## Abstract

Reflective thinking is crucial for academic success, personal development, and cultural adaptation. Therefore, this study aimed to analyze the role of the Reflective Thinking Scale (RTS) for international students in Chinese universities. Data were collected from 482 international students in Northwestern China. Exploratory Factor Analysis (EFA), Confirmatory Factor Analysis (CFA), Reliability, and Correlation Analysis were employed to validate the RTS. The qualitative phase used semi-structured interviews and reflective journals. The results revealed a four-factor structure for the RTS: habitual action, understanding, reflection, and critical reflection. The scale showed strong reliability (Cronbach’s α = 0.77) with significant positive correlations between reflective thinking and academic performance. The qualitative phase drew on 26 interviews and 22 reflective journals, analyzed thematically. Six themes emerged, highlighting reflection’s role in academic regulation, language acquisition, cultural adaptation, emotional processing, and personal identity development. The findings confirm the cross-cultural validity of the RTS and underscore the importance of scaffolded reflective practices in international education. This study also extends theoretical links between reflective thinking, transformative learning, cognitive flexibility, and self-regulated learning. Implications are offered for educators, curriculum designers, and researchers seeking to enhance reflective learning environments for diverse student populations.

## 1. Introduction

Reflective thinking, the process of critically examining thoughts, actions, and experiences, is essential for effective learning, problem-solving, and professional development. John Dewey’s (1933) seminal work indicated that reflective thinking is an active, persistent, and deliberate process that fosters deeper understanding and boosts decision-making. This foundational understanding has shaped student-centered learning and critical pedagogy (Shaffer, 2015). Similarly, Donald Schön (1983) introduced reflection-in-action and reflection-on-action, which highlight the dynamic nature of reflective practice. Reflection-in-action refers to adjusting actions in real-time, while reflection-on-action involves evaluating and learning from experiences afterward. These concepts have been influential in fields like teacher education, where reflective practice is essential for continuous professional growth (Ottesen, 2007). From this perspective, reflective thinking serves as a bridge between theory and practice, due to which it has gained attention for its role in enhancing self-regulated learning, metacognitive awareness, and cognitive flexibility worldwide (Zimmerman, 2000; Orakcı, 2021; Afshar & Farahani, 2015). It supports learners in long-term planning, monitoring, and evaluating their progress, enabling them to adapt strategies and respond to academic and emotional challenges. The importance of reflection is even greater for international students, who face academic pressures, linguistic barriers, and cultural adaptation in unfamiliar educational contexts (Guo et al., 2022; W. Liu & Rathbone, 2021).

Globally, reflective thinking is viewed as a vital human experience, which provides opportunities to make sense of one’s life or curricular experiences. With the trend of globalization in education, more individuals choose to study abroad to have a deeper understanding of different cultures. The Reflective Thinking Scale (RTS), developed by Kember et al. (2000), is one of the most widely used tools for measuring reflective engagement. It categorizes reflective thinking into four dimensions: habitual action, understanding, reflection, and critical reflection. Although having been validated in various academic and professional fields (Basol & Evin Gencel, 2013; Mourão et al., 2022), its applicability to international students in Chinese universities has not been sufficiently examined. Cultural paradigms influence how reflection is expressed; in collectivist societies like China, reflective behavior may be more relational and socially embedded compared to the introspective and individualist style seen in Western settings (Lee, 2017; Martin & Nakayama, 2021).

Many previous studies have been conducted about how reflective thinking scales promote mindfulness, academic achievement, and identity formation (Choy et al., 2019; Aydoğmuş & Şentürk, 2023; Hammad Al-Rashidi & Aberash, 2024; Gong et al., 2024). Hammad Al-Rashidi and Aberash (2024) studied the Reflective Thinking Scale’s applicability for international students in China, confirming a modified version suitable for non-native learners through exploratory and confirmatory factor analysis. Their findings highlighted the scale’s effectiveness in assessing metacognitive engagement in multicultural academic settings. Sabariego Puig et al. (2020) explored reflective thinking dimensions among university students with factor analysis, providing statistical support for the scale’s international adaptation, emphasizing the need for context-specific validation. Gong et al. (2024) focused on international students’ experiences with reflective learning tools in Chinese higher education, revealing cultural and linguistic influences on their responses to the scale and stressing the need for culturally sensitive adjustments in measurement instruments. Aydoğmuş and Şentürk (2023) found that reflective thinking training significantly improves university students’ learning motivation and responsibility. Similarly, Chamdani et al. (2022) demonstrated a strong correlation between reflective engagement and academic performance. Despite these findings, the construct of reflective thinking remains underexplored in non-Western educational contexts, particularly among international students in China (An & Chiang, 2015; Colomer et al., 2020). From the previous literature, there was no detailed study about the role of the Reflective Thinking Scale for international students in China.

In this regard, this study addresses this research gap by validating the RTS for international students enrolled in Chinese universities and examining the role reflective thinking plays in their academic and personal development by using a mixed-methods design. This method integrates factor analysis and qualitative thematic exploration. This research would be an essential process in the improvement of critical thinking skills. Critical thinking involves complex mental processes, higher-order thinking abilities, or cognitive skills addressed in educational reforms.

### 1.1. Significance of the Reflective Thinking Scale

The importance of reflective thinking is well recognized in Western contexts, but its application and measurement in non-Western settings remain underexplored, particularly for international students in China. With over 492,000 international students enrolled in Chinese universities by 2018 (China Ministry of Education, 2019), there is an urgent need for culturally relevant tools to assess and foster reflective thinking. Although the Reflective Thinking Scale (RTS) developed by Kember et al. (2000) is widely validated, its applicability to international students in Chinese universities remains under-researched.

In addition, reflective thinking is crucial for meaningful learning and metacognitive development. It helps learners monitor cognitive processes, regulate behaviors, and assess academic performance (Zimmerman, 2000; Orakcı, 2021). This is especially vital for international students adjusting to new educational and cultural contexts. Reflection enables students to navigate diverse perspectives, overcome language barriers, and build culturally relevant knowledge (An & Chiang, 2015; Guo et al., 2022). The Reflective Thinking Scale (RTS) by Kember et al. (2000) is a prominent tool for measuring reflective engagement, identifying four levels: habitual action, understanding, reflection, and critical reflection. The RTS shows psychometric validity in various areas, including education (Basol & Evin Gencel, 2013), healthcare (Liao & Wang, 2019), and professional development (Mourão et al., 2022). However, few studies have assessed its validity among diverse non-Western and multilingual populations (An & Chiang, 2015; Guo et al., 2022). Validating the RTS is essential for ensuring its relevance and accuracy. Given the rise of international students in Chinese universities and cultural influences on reflection, evaluating the RTS is both theoretically and practically significant (Lee, 2017; W. Liu & Rathbone, 2021). Tools validated in one culture may not fully reflect the tendencies of learners from diverse backgrounds. This study aims to explore the RTS’s structure and applicability among international students in China.

### 1.2. Objectives of the Study

This study aims to fill this gap by validating the RTS for international students at Shaanxi Normal University (SNNU) and Henan University (HENU) in Northwestern China. This study contributes to the literature by validating the RTS for international students in China, providing insights into how reflective thinking supports their academic achievement and personal growth in a non-Western educational setting. By integrating both quantitative and qualitative methods, this study’s aim is twofold: First, it aims to validate the Reflective Thinking Scale (RTS) among international students in Chinese universities by examining its psychometric structure and internal consistency. Second, it seeks to explore how reflective thinking is used by these students to manage academic challenges, cultural transitions, and personal development in a multilingual and multicultural educational environment. These objectives are addressed through the following research questions:

1. Does the RTS demonstrate valid factor structure and internal reliability when applied to international students in China?
2. What is the role of reflective thinking in their academic and cultural adjustment among international students?
3. What themes emerge from students’ reflections that illustrate cognitive, emotional, and behavioral adaptation?

## 2. Literature Review

Reflective thinking has long been recognized as a critical component of effective learning and professional development. It involves the process of actively and deliberately examining one’s experiences, actions, and beliefs (John Dewey, 1933). Reflective thinking promotes a deeper understanding of subject matter, better decision-making, and the development of critical thinking skills. In recent years, reflective thinking has gained increased attention in the educational field, particularly in relation to its role in enhancing problem-solving skills and learning strategies (Aydoğmuş & Şentürk, 2023; Chamdani et al., 2022). This literature review section presents a comprehensive overview of key theoretical models, empirical findings, and assessment frameworks. It begins with foundational theories that define the concept and its dimensions, followed by a review of how reflective thinking influences academic outcomes and metacognitive growth. The next section introduces the RTS, its psychometric structure, and its use across various fields. Finally, the review explores how reflective practices operate among international students, with attention to cultural framing and emotional adaptation. Together, these strands provide the conceptual and empirical basis for validating the RTS and investigating reflective engagement in the context of international higher education in China.

### 2.1. Concept of Reflective Thinking

The concept of reflective thinking was first thoroughly introduced by John Dewey (1933), who described it as an active, persistent, and careful consideration of any belief, scrutinized. Reflective thinking is an essential cognitive process that allows learners to critically evaluate experiences and extract meaning from them. This emphasis on structured thought also aligns with Bloom’s (1956) taxonomy of educational objectives, which categorizes learning outcomes from basic knowledge to higher-order reflection and evaluation. This foundational idea has influenced educational practice by highlighting reflection as a deliberate and structured mode of inquiry. Dewey’s work established reflective thinking as an active process of constructing knowledge applicable to real life (John Dewey, 1933). This perspective influences educational practices, particularly critical pedagogy and student-centered learning (Shaffer, 2015). Donald Schön (1983) further developed this by introducing reflection-in-action and reflection-on-action, highlighting the dynamic nature of reflective practice, especially in professional contexts like teacher education, where these models enhance teaching and learning outcomes (Aslam et al., 2021; Amalia et al., 2025). Reflection-in-action is spontaneous during events, while reflection-on-action is retrospective, leading to deeper insights. Boud et al. (1985) emphasized turning experiences into learning through emotional and cognitive engagement, applicable in multicultural settings to navigate ambiguity and identity (Brookfield, 1988; W. Liu & Rathbone, 2021). Mezirow’s (1991) theory of transformative learning framed reflection as a means to critically reassess assumptions and develop new perspectives. This allows learners to adapt epistemologically in response to complex experiences. In modern education, reflective thinking is considered a multidimensional process that fosters metacognitive awareness, emotional regulation, and adaptive learning (Zimmerman, 2000; Orakcı, 2021). Afshar and Farahani (2015) found that students with strong reflective skills exhibited higher emotional intelligence and academic success. Reflective learners tend to evaluate and adjust their strategies, enhancing engagement and performance in various academic contexts (Gong et al., 2024).

### 2.2. Linkage of Reflective Thinking with Learning Outcomes

The relationship between reflective thinking and learning outcomes has been widely explored in educational research. Aydoğmuş and Şentürk (2023) found that reflective thinking is a significant predictor of effective learning strategies. This aligns with Chamdani et al. (2022), who demonstrated that students who engage in reflective thinking exhibit higher academic achievement due to better engagement with learning materials and more effective study habits. Furthermore, reflective thinking promotes metacognitive awareness, enabling students to plan, monitor, and evaluate their learning processes (Zimmerman, 2000). This metacognitive aspect of reflective thinking has been linked to self-regulated learning (Kapranos, 2007). Guo et al. (2022) explored that reflective thinking positively influences students’ epistemological beliefs, which in turn impacts their academic success in STEM education. Recent studies have demonstrated that reflective thinking helps students not only to solve problems but also to develop creative solutions by evaluating and rethinking their actions (Saritepeci & Yildiz Durak, 2024; C. Liu et al., 2023). In this way, reflective thinking contributes to the development of higher-order thinking skills, essential for navigating complex tasks and adapting to new contexts (C. Liu et al., 2023).

Moreover, reflective thinking empowers learners to assess strategies, manage cognitive tasks, and track academic progress. Zimmerman (2000) emphasizes that reflection is fundamental to self-regulated learning, involving goal-setting, performance evaluation, and strategy modification. Kolb’s (1984) experiential learning theory recognizes reflection as a crucial phase that helps learners turn experiences into abstract concepts, thus enhancing learning efficiency and autonomy. In educational psychology, reflective thinking promotes engagement and boosts academic success. Chamdani et al. (2022) found a strong link between students’ reflective skills and performance, particularly in time management and motivation. In language learning, Guo et al. (2022) indicated that reflective engagement aids multilingual learners’ and comprehension. Ural and Dadli (2020) reported that reflective learners display better vocabulary retention and oral fluency. Additionally, W. Liu and Rathbone (2021) noted that international students in China practicing reflective journaling achieved improved academic writing and cultural integration.

### 2.3. The Reflective Thinking Scale (RTS)

One of the most widely used instruments for measuring reflective thinking is the Reflective Thinking Scale (RTS), developed by Kember et al. (2000). The RTS measures reflective thinking across four dimensions: habitual action, understanding, reflection, and critical reflection. These dimensions represent a continuum of reflective thinking, with habitual action representing the least reflective level and critical reflection representing the most advanced form. RTS has been validated across various educational fields. Basol and Evin Gencel (2013) confirmed its reliability in teacher education, while Mourão et al. (2022) adapted it for professional training. The RTS has been validated across various contexts, including social sciences (Leung & Kember, 2003; Aslam et al., 2021) and healthcare education (Liao & Wang, 2019), demonstrating its robustness in assessing reflective thinking. Despite its widespread use, there has been limited research on the cultural applicability of the RTS. While the RTS has been validated in Western contexts, there is a gap in understanding how reflective thinking is practiced and measured among international students in Chinese universities. China has become a prominent destination for international students, with over 492,000 international students enrolled in 2018 (China Ministry of Education, 2019). Therefore, it is essential to validate tools like the RTS to ensure that they accurately assess reflective thinking in this culturally diverse population. Research has shown that students from collectivist cultures may focus more on the social implications of their actions, while those from individualistic cultures may prioritize personal growth (Guo et al., 2022). However, there is limited research on the RTS in multilingual or non-Western contexts, especially among international students in Asia. Given that reflection is influenced by cultural norms, testing the RTS’s validity in diverse environments is crucial. This study aims to validate the RTS for international students in Chinese universities.

### 2.4. Role of Reflective Thinking Among International Students

Reflective thinking has been shown to help students navigate these challenges by allowing them to assess their experiences critically (Guo et al., 2022). Recent research by Hammad Al-Rashidi and Aberash (2024) demonstrated that reflective thinking plays a significant role in the personal and academic growth of EFL students by improving their mindfulness, resilience, and academic well-being. For international students in China, reflective thinking can help them adapt to the academic culture, enhance their language proficiency, and develop cross-cultural competencies (Gong et al., 2024). Reflective thinking provides these students with the tools to evaluate their learning strategies, adjust to new academic expectations, and manage the cultural stress often associated with studying abroad (Aydoğmuş & Şentürk, 2023). However, there is a need for more research that specifically addresses how reflective thinking tools like the RTS can be used effectively with international students in non-Western educational contexts, particularly in China.

Reflective thinking is crucial for international students as they navigate academic adjustments, language learning, and cultural integration. They face challenges that necessitate adapting to new communication norms and classroom expectations. Reflection aids in assessing experiences and monitoring adaptation (Colomer et al., 2020; Guo et al., 2022). In collectivist cultures like China, reflection emphasizes social harmony over individual critique (Lee, 2017; Martin & Nakayama, 2021). This cultural perspective influences how reflective tasks are approached in classrooms (Kim et al., 2021; Park et al., 2022). Jin and Cortazzi (2013) highlight that students from Confucian backgrounds view reflection as a means to enhance group cohesion. Martin and Nakayama (2021) state that effective intercultural communication requires ongoing identity negotiation, indicating the need for reflective awareness when managing cultural expectations. Additionally, reflective thinking promotes emotional growth, improving students’ emotional regulation and resilience, vital for handling cross-cultural transitions (Yaacob et al., 2021; Afshar & Farahani, 2015). Digital storytelling supports emotional processing in online intercultural settings (Kim et al., 2021). Hence, reflection serves as a key academic skill and personal growth tool.

### 2.5. Theoretical Foundations of Reflective Thinking

The current study utilizes three significant theoretical frameworks to examine the function of reflective thinking in international education: transformative learning theory, cognitive flexibility theory, and self-regulation theory. Mezirow’s (1991) transformative learning theory defines reflection as a process by which learners critically evaluate their assumptions, particularly in response to novel or disorienting experiences. Through this critical reflection, individuals can progress beyond superficial understanding and undergo perspective transformation, a crucial process for intercultural learning and identity formation. Likewise, Brookfield (1988) described critical reflection as a means for learners to challenge dominant assumptions, which is vital for personal growth and transformation across diverse educational environments. This perspective aligns with the RTS component of critical reflection, which encompasses evaluative thinking that has the potential to alter values and beliefs.

**Figure 1:**
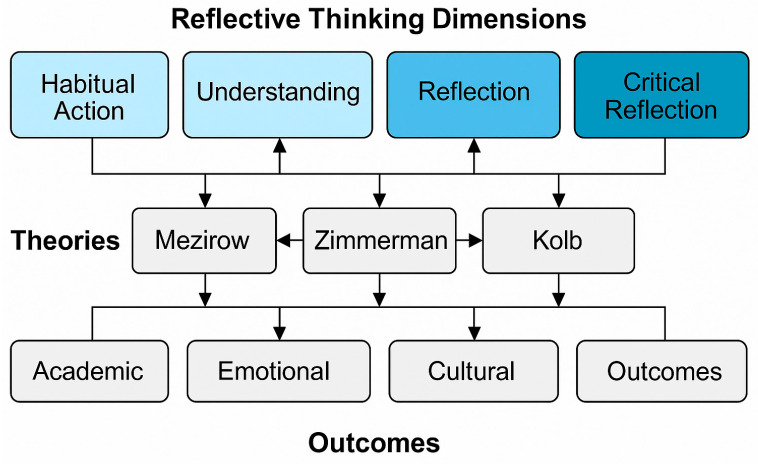
Conceptual framework connecting reflective thinking dimensions with theoretical foundations and outcome domains.

Reflective thinking serves as a fundamental element of cognitive flexibility according to Spiro et al. (1987), allowing learners to modify their viewpoints, adapt their existing knowledge, and incorporate insights that are specific to contexts. For international students in culturally varied academic settings, cognitive flexibility enhances their ability to engage in adaptive learning and adjust to diverse sociocultural environments. Zimmerman’s (2000) self-regulation theory highlights the cyclical nature of goal establishment, self-evaluation, and strategy modification. Reflection acts as a metacognitive mechanism that enables students to evaluate their learning approaches, manage their motivation, and improve their performance strategies. Research outcomes (Afshar & Farahani, 2015; Chamdani et al., 2022) indicate that reflective thinking plays a significant role in students’ capacity to endure, adapt, and thrive amid various educational challenges. Collectively, these theoretical models offer a comprehensive framework for comprehensively understanding the multifaceted impact of reflection on academic adaptation, cultural assimilation, and personal growth in international students. These theoretical insights are incorporated into a conceptual model that associates reflective thinking with outcomes for cross-cultural students (Figure 1).

This model incorporates four essential elements of reflective thinking: habitual action, understanding, reflection, and critical reflection, alongside theoretical frameworks such as transformative learning, cognitive flexibility, and self-regulation. Arrows are used to demonstrate how these elements contribute to academic adjustment, emotional regulation, and cultural adaptation in international students. Despite extensive theoretical and measurement work on reflective thinking, most empirical studies have largely occurred within Western or monocultural contexts. Research validating reflective thinking instruments, particularly the Reflective Thinking Scale, is notably scarce for international students within culturally diverse, non-Western academic environments. Additionally, there are few studies that combine quantitative validation with qualitative inquiry to explore the role of reflective thinking in academic adaptation, emotional regulation, and identity development. This illustrates the necessity for research that is sensitive to cultural differences and captures both the structural validity of the RTS and the lived experiences of those engaged in reflective practices. To address this gap, the current study utilizes a sequential explanatory mixed-methods design that merges factor analysis with thematic analysis of interviews and reflective journals, facilitating a deeper understanding of reflective thinking across cognitive, emotional, and cultural dimensions.

## 3. Methodology

This study utilizes a mixed-methods approach to investigate the role of reflective thinking in academic success and personal growth among international students in China. By combining quantitative and qualitative methods, this research aims to provide a comprehensive understanding of how reflective thinking impacts student outcomes. The quantitative component focuses on validating the Reflective Thinking Scale (RTS), while the qualitative component explores student experiences through interviews and reflective journals. The methodology is organized into four components: research design, participants, data collection, and data analysis.

### 3.1. Research Design

This study employed a sequential explanatory mixed-methods design, as described by Creswell and Plano Clark (2017), to examine both the structural validity of the Reflective Thinking Scale (RTS) and the reflective experiences of international students studying in China. The sequential nature of the design enabled the qualitative findings to build upon and contextualize the quantitative results. By integrating these two strands of data, this study ensured both statistical generalizability and contextual depth, which are critical for understanding reflective thinking as a culturally embedded construct. The design consisted of two distinct phases. In the quantitative phase, statistical methods were used to explore and confirm the factor structure of the RTS, and to assess its reliability and internal consistency. In the qualitative phase, data were collected from semi-structured interviews and weekly reflective journals to provide deeper insight into how students used reflection to navigate academic and cultural challenges.

### 3.2. Research Participants

The sample comprised 482 international students from Shaanxi Normal University (SNNU) and Henan University (HENU) in China, selected for their large international student populations and cultural diversity. A convenience sampling method was used, with participation being voluntary and confidential. The participants were enrolled in both undergraduate and postgraduate programs across 50 academic disciplines, ensuring a broad range of academic fields and cultural backgrounds. The participants were taken from 12 different countries, including Pakistan, Yemen, Egypt, Australia, Sudan, Ethiopia, Iraq, Russia, Nigeria, Cameroon, Japan, and Rwanda. The sample included both undergraduate and postgraduate students across various disciplines such as education, business, engineering, and health sciences. For the qualitative phase, 26 students were selected for semi-structured interviews, and 22 students submitted weekly reflective journals over a six-week period. Selection was based on voluntary response sampling from those who completed the survey. The sample size for the quantitative phase (*n* = 482) exceeds the recommended threshold for conducting exploratory and confirmatory factor analyses in structural equation modeling (Wolf et al., 2013), ensuring sufficient statistical power. Detailed participant demographics, including gender, academic level, and age range, are presented in Table 1.

### 3.3. Data Collection

Data collection occurred in two phases, including a quantitative and a qualitative phase. In the quantitative phase, data were collected using an online survey featuring the Reflective Thinking Scale (RTS) (Appendix A). The RTS is categorized into a 16-item self-report questionnaire measuring reflective thinking across four dimensions: habitual action, understanding, reflection, and critical reflection (Kember et al., 2000). Participants rated each statement on a 5-point Likert scale, ranging from 1 (“strongly disagree”) to 5 (“strongly agree”). The total score, ranging from 16 to 80, reflects the level of reflective thinking, with higher scores indicating greater reflection. The survey was distributed through WeChat among international students in China, ensuring convenient access. Ethical guidelines were strictly followed through confidentiality and voluntary participation.

The qualitative phase involved semi-structured interviews and reflective journals to explore students’ reflective thinking processes, experiences, and challenges within the Chinese academic context. In this phase, two types of data were collected. First, 26 students participated in semi-structured interviews lasting between 30 and 45 min, conducted in person or through video conferencing tools. Interview questions focused on students’ reflective practices, academic coping strategies, and experiences navigating cultural challenges. Second, 22 participants submitted weekly reflective journals over a six-week period. Each journal included responses to structured prompts addressing academic setbacks, emotional responses, adaptation strategies, and self-reflective learning (Walker, 2006). Participation in all phases of the study was voluntary and conducted with full informed consent.

#### Ethical Considerations

This study was conducted in accordance with the Declaration of Helsinki and approved by the Institutional Review Board (IRB) of Shaanxi Normal University (protocol code SNNU-IRB-2023-045, date of approval: 15 March 2023) and Henan University (protocol code HENU-IRB-2023-132, date of approval: 20 September 2023). Ethical approval was obtained for all procedures involving human participants, ensuring compliance with ethical standards. Confidentiality and anonymity were maintained throughout the research process.

Informed consent was obtained from all participants involved in the study. Participants were provided with detailed information about the study’s purpose, procedures, and their rights, including the right to withdraw at any time without consequences. Written informed consent was secured prior to participation, ensuring voluntary and informed involvement. No personally identifiable information was collected, and all data were anonymized for analysis.

### 3.4. Data Analysis

The quantitative data collected through the Reflective Thinking Scale (RTS) were analyzed using SPSS (v26) and AMOS (v24) software. Exploratory Factor Analysis (EFA) identified the underlying structure of the RTS. Sampling adequacy was assessed using the Kaiser–Meyer–Olkin (KMO) index and Bartlett’s Test of Sphericity. Next, a Confirmatory Factor Analysis (CFA) was performed to validate the factor structure, and model fit was evaluated using standard indices: χ^2^/df ratio (acceptable ≤ 3), RMSEA (<0.08), SRMR (<0.08), CFI and TLI (≥0.90). Cronbach’s alpha coefficients were calculated for each factor, with values of α ≥ 0.70 deemed acceptable for internal consistency (Nunnally, 1994).

Pearson correlation coefficients were calculated to assess the relationship between overall reflective thinking and self-rated academic performance, contributing to Research Question 1. In the qualitative phase, thematic analysis was conducted on interview transcripts and reflective journals using Braun and Clarke’s (2006) six-phase approach. Initial codes were developed inductively and then organized into higher-order themes based on recurrence and relevance to the research aims. Thematic development was guided by Research Questions 2 and 3, which explored how students described the role of reflective thinking in academic and cultural adaptation, and what patterns emerged across emotional, cognitive, and behavioral dimensions.

## 4. Results

This section highlights the outcomes of both the quantitative and qualitative phases, following the sequential explanatory mixed-methods design. The quantitative phase addressed Research Question 1, which focused on validating the RTS through exploratory and confirmatory factor analysis, reliability testing, and correlation analysis. The qualitative phase addressed Research Questions 2 and 3, exploring how international students described the role of reflective thinking in their academic and cultural adjustment and identifying key themes related to their cognitive, emotional, and behavioral adaptation. The results are documented in two main sections: (1) quantitative-based analysis of the RTS and its psychometric properties, and (2) thematic findings based on interview transcripts and reflective journals.

### 4.1. Quantitative Analysis

The quantitative stage of this research sought to validate the structure, reliability, and predictive validity of the Reflective Thinking Scale (RTS) among international students enrolled in Chinese universities. This analysis specifically addressed Research Question 1, which aimed to investigate the psychometric characteristics of the RTS through various statistical methods. Initially, Exploratory Factor Analysis (EFA) was employed to identify the scale’s underlying factor structure. Subsequently, Confirmatory Factor Analysis (CFA) assessed the fit of the identified factor model. The internal consistency of the scale was measured using Cronbach’s alpha, while test–retest reliability was assessed to evaluate temporal stability. Furthermore, Pearson’s correlation analysis was conducted to explore the relationship between reflective thinking and academic performance, and independent samples t-tests were utilized to determine differences in RTS scores based on gender and academic level. The results from these analyses are detailed in the subsequent subsections (Section 4.1.1, Section 4.1.2, Section 4.1.3, Section 4.1.4, Section 4.1.5 and Section 4.1.6).

#### 4.1.1. Exploratory Factor Analysis (EFA)

An Exploratory Factor Analysis (EFA) was conducted to identify the underlying structure of the Reflective Thinking Scale (RTS). The Kaiser–Meyer–Olkin (KMO) measure of sampling adequacy was 0.79, indicating that the data were highly suitable for factor analysis. Additionally, Bartlett’s Test of Sphericity yielded a significant result (χ^2^ = 11,634.93, *p* < 0.001), confirming that the correlation matrix was factorable and appropriate for further analysis. The EFA revealed a four-factor structure consistent with the original RTS model proposed by Kember et al. (2000). These factors—habitual action, understanding, reflection, and critical reflection—collectively explained 67.526% of the total variance. The factor loadings ranged from 0.584 to 0.877, while the communalities ranged from 0.399 to 0.834, indicating strong relationships between the items and their respective factors. These results suggest that the RTS is a robust tool for measuring reflective thinking in the context of international students in China.

**Figure 2:**
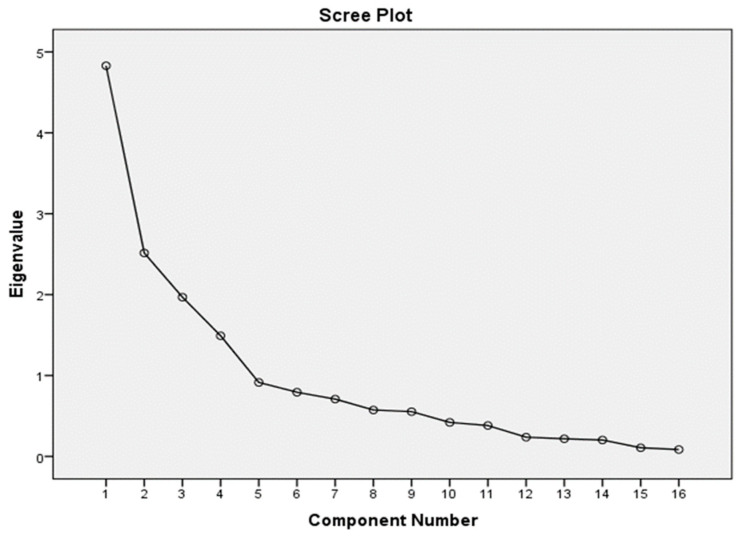
Scree plot from exploratory factor analysis.

To further validate the factor structure, principal component analysis (PCA) was employed. The KMO value of 0.716 confirmed the adequacy of the sample for factor analysis, as values above 0.7 are considered acceptable (Field, 2009). The significance of Bartlett’s Test of Sphericity (BST) (χ2 = 4430.769, *p* < 0.001) further supported the factorability of the data, indicating a normal distribution of the variables. These findings align with the recommendations of Kaiser (1974), who suggested that a KMO value greater than 0.5 is suitable for factor analysis. The results of the EFA and PCA collectively demonstrate the appropriateness of the data for factor analysis and the robustness of the four-factor model. Figure 2 indicates the scree plot, which visually represents the eigenvalues for each factor, confirming the optimal number of factors to retain (four factors).

#### 4.1.2. Factor Loadings and Communalities of the RTS

The Kaiser–Meyer–Olkin (KMO) measure of sampling adequacy was reported at 0.716, indicating that the data are suitable for factor analysis (Kaiser, 1974; Field, 2009). Additionally, Bartlett’s Test of Sphericity yielded a significant result (χ^2^ = 4430.769, *p* < 0.001), which further supports the factorability of the correlation matrix. These findings suggest that the Reflective Thinking Scale (RTS) is suitable for factor analysis. Detailed in Table 2 are the factor loadings and communalities for each item. The four identified factors are: Habitual Action (Factor 1), which comprises four items with factor loadings between 0.584 and 0.877; Understanding (Factor 2), which consists of three items with loadings ranging from 0.638 to 0.910; Reflection (Factor 3), which includes four items with loadings between 0.584 and 0.877; and Critical Reflection (Factor 4), which features four items with factor loadings from 0.739 to 0.854. The communalities for all items vary from 0.399 to 0.834, reflecting strong correlations between the items and their corresponding factors.

#### 4.1.3. Correlation Among Factors

Table 3 shows the correlation matrix for the four factors. All factors were significantly correlated (*p* < 0.01), with correlation coefficients ranging from 0.429 to 0.905. According to Heale and Twycross (2015), correlations above 0.5 indicate strong associations, confirming the structural validity of the RTS (Table 4).

The scree plot displays a clear elbow after the fourth factor, supporting the four-factor solution retained for the RTS. The EFA results confirm the theoretical four-factor structure of the RTS as proposed by Kember et al. (2000). Each factor is supported by strong and distinct loadings, and the communalities demonstrate that all retained items contribute meaningfully to the latent constructs. The variance explained and the scree plot collectively validate the four-factor retention.

#### 4.1.4. Inter-Factor Correlation Analysis

To examine the relationships among the four reflective thinking components, Pearson’s correlation coefficients were calculated. The results revealed positive and statistically significant correlations among all subscales (*p* < 0.01), indicating that while conceptually distinct, the components of reflective thinking are moderately to strongly related. The correlation coefficients ranged from 0.429 to 0.905, suggesting that individuals who scored high on one dimension of reflective thinking were also likely to score higher on others. These findings support the internal coherence of the RTS and are consistent with previous research on reflective constructs (Kember et al., 2000). According to Heale and Twycross (2015), correlations above 0.5 indicate strong associations, confirming the structural validity of the RTS (Table 4).

The highest correlation was observed between Reflection and Critical Reflection (r = 0.905), suggesting a close conceptual link between reflective depth and critical evaluation. The lowest, yet still significant, correlation was between Habitual Action and Critical Reflection (r = 0.429), consistent with the idea that habitual responses may inversely relate to deeper reflection processes.

#### 4.1.5. Internal Consistency and Test–Retest Reliability

The reliability of the Reflective Thinking Scale (RTS) was assessed using two indicators: internal consistency via Cronbach’s alpha and temporal stability via test–retest reliability. Cronbach’s alpha coefficients for the four RTS dimensions ranged from 0.67 to 0.77, with an overall alpha of 0.77, indicating acceptable internal consistency (Nunnally, 1994). These values suggest that the items within each subscale are consistently measuring their intended constructs. Test–retest reliability was assessed by administering the RTS to a subsample of participants at two different time points, separated by two weeks. The resulting coefficients ranged from 0.73 to 0.83, with an overall test–retest reliability of 0.83, confirming the instrument’s stability over time (see Table 5).

These results demonstrate that the RTS is both internally consistent and stable over time, validating its use in cross-cultural educational settings and longitudinal research. While the Cronbach’s alpha values for Habitual Action and Understanding are slightly below 0.70, they are still acceptable given the exploratory nature of the study and the context-specific adaptation of the instrument.

#### 4.1.6. Confirmatory Factor Analysis (CFA)

**Figure 3:**
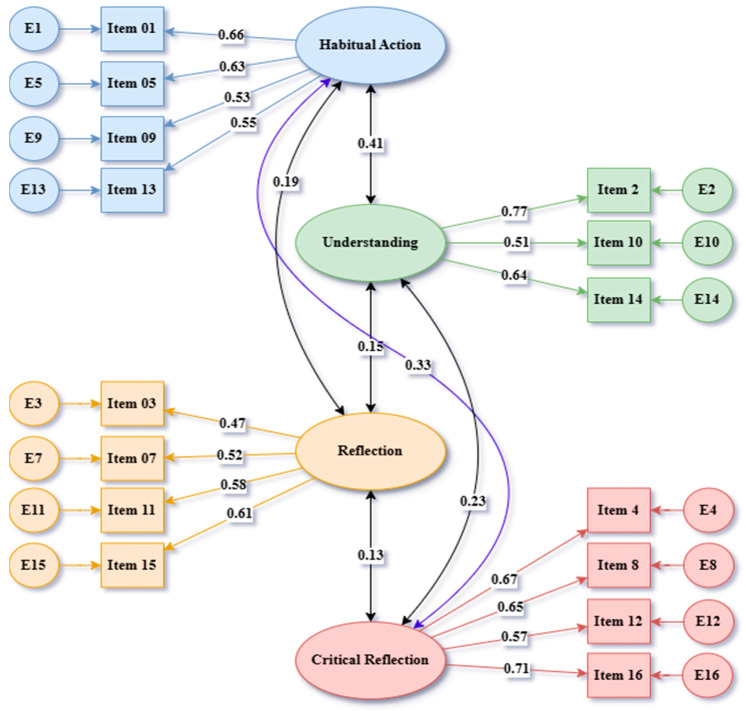
Confirmatory factor analysis (CFA) model for the RTS.

A Confirmatory Factor Analysis (CFA) was performed using AMOS 24.0 to validate the four-factor structure that emerged during the Exploratory Factor Analysis (EFA) phase. The model consisted of 15 items associated with four latent constructs: Habitual Action, Understanding, Reflection, and Critical Reflection. This analysis specifically addressed Research Question 1, which focused on the structural validity of the RTS among international students. The results of the CFA indicated an acceptable model fit, as detailed in Table 6. The ratio of chi-square to degrees of freedom (χ^2^/df) was 4.061, which is within the acceptable range for large samples. The Comparative Fit Index (CFI = 0.901), Goodness-of-Fit Index (GFI = 0.900), and Tucker–Lewis Index (TLI = 0.910) all exceeded the 0.90 benchmark, demonstrating a good model fit. Additionally, the Root Mean Square Error of Approximation (RMSEA = 0.073) was also within acceptable parameters (<0.08). These findings confirm the validity of the RTS structure across a multicultural sample. The standardized path diagram is illustrated in Figure 3. All standardized factor loadings were statistically significant (*p* < 0.001) and ranged between 0.62 and 0.86, indicating strong relationships between the observed items and their corresponding latent constructs. These findings provide strong support for the structural validity of the RTS in international academic settings.

The final RTS consists of 15 items distributed across four factors: (1) Habitual Action, which includes four items; (2) Understanding, with three items; (3) Reflection, comprising four items; and (4) Critical Reflection, which includes four items, as illustrated in Figure 3.

To examine differences in reflective thinking based on demographic variables, independent samples t-tests were conducted using total RTS scores across gender and academic level. The results are presented in Table 7. A statistically significant difference was observed by gender, with male students reporting higher reflective thinking scores (t(480) = 5.34, *p* < 0.001). Similarly, postgraduate students scored significantly higher than undergraduates (t(480) = 4.57, *p* < 0.001). These findings may reflect influences related to academic maturity, life experience, and cognitive engagement.

### 4.2. Qualitative Results

The qualitative aspect of this research focused on examining the experiences and perceptions of international students regarding reflective thinking within both their academic and cultural contexts. Data were gathered through semi-structured interviews and reflective journals and were then analyzed using thematic analysis to identify significant themes and sub-themes. These themes provide insights into the impact of reflective thinking on students’ educational experiences, academic achievements, and personal development. The subsequent sections present a summary of the primary findings, emphasizing the significance of reflective thinking in navigating cultural, linguistic, and academic challenges. The key themes derived from the interviews and journals indicate that reflective thinking is crucial for academic achievement, cultural adjustment, language development, and problem-solving. It assists students in gaining a deeper understanding of their learning processes, modifying their strategies, and fostering personal and academic advancement. A detailed summary of the qualitative results can be found in Table 8.

1. **Cultural Adaptation through Reflective Awareness**

Participants described reflection as a strategy for decoding unfamiliar social and academic norms. It facilitated cultural sensitivity and improved classroom participation.

*“Reflecting on my interactions with Chinese classmates helped me understand their communication style, which was very different from my own.”*

Male undergraduate, Pakistan, Engineering (Interview 4).

*“At first, I felt lost in the classroom, but after reflecting, I began adjusting how I communicated and participated.”*

Female undergraduate, Rwanda, Biology (Journal 8).

These findings affirm the role of reflection in intercultural competence, especially in collectivist academic contexts.

2. **Language Development through Metacognitive Journaling**

Reflective writing helped students monitor language acquisition and develop targeted strategies for improvement.

*“I realized I needed to focus more on speaking than writing—it came out through my reflections.”*

Female postgraduate, Ethiopia, Linguistics (Journal 3).

*“In my journal, I started recording new words weekly and made an effort to use them in class.”*

Male undergraduate, Nigeria, Economics (Journal 10).

This aligns with Zimmerman’s (2000) model of metacognitive self-regulation.

3. **Strategic Academic Adjustment and Self-Regulation**

Students identified and adjusted ineffective study habits through reflection.

*“I started using spaced repetition instead of passive reading after reflecting on my exam failures.”*

Male undergraduate, Nigeria, Business (Interview 6)

*“Reflecting showed I was overloading myself in one subject—I restructured my study plan.”*

Female postgraduate, Yemen, Public Health (Journal 5)

These outcomes reinforce reflection’s role in academic resilience and time management.

4. **Need for Structured Reflective Scaffolding**

Despite the benefits, many participants struggled with how to reflect deeply and sought clearer institutional support.

*“Our professors just told us to reflect—but how? I needed more guidance.”*

Female undergraduate, Yemen, Education (Interview 7)

*“The prompts helped, but I still wasn’t sure if I was doing it right.”*

Male postgraduate, Cameroon, Software Engineering (Journal 12)

These experiences support Q. Liu et al. (2025), who emphasize the importance of scaffolded reflection models.

5. **Personal Growth and Identity Formation**

The journals revealed a transformation in students’ self-concept, values, and confidence.

*“Each journal entry helped me understand how much I’ve matured since arriving in China.”*

Female postgraduate, Russia, International Relations (Journal 17)

*“Initially, I felt helpless, but my reflections helped me become more resilient.”*

Male undergraduate, Egypt, Environmental Science (Interview 9)

This reflects Mezirow’s (1991) theory of transformative learning.

6. **Emotional Regulation and Interpersonal Dynamics**

Reflection helped students process emotions and manage peer interactions constructively.

*“After reflecting on a conflict in a group project, I approached the next one differently—more calmly.”*

Female undergraduate, Iraq, Sociology (Interview 11)

*“Writing helped me process frustration and adjust how I communicate with peers.”*

Male postgraduate, Sudan, Computer Science (Journal 15).

Reflection thus served as a mechanism for emotional intelligence and social awareness.

## 5. Discussion

This section combines the quantitative and qualitative results of the study to analyze the manifestation of reflective thinking among international students in Chinese universities. The discussion is structured thematically, focusing on important relationships identified in the study: academic performance, cultural adaptation, language acquisition, and personal growth. The analysis establishes that reflective thinking is a multidimensional concept that has substantial effects on the academic success and adaptation of international students. The synthesis of statistical findings and participant narratives offers a comprehensive insight into how students utilize reflection as a learning strategy, a means of cultural adjustment, and a method for personal development.

### 5.1. Reflective Thinking and Academic Performance

The quantitative results indicated statistically significant positive correlations among all four components of reflective thinking—habitual action, understanding, reflection, and critical reflection and students’ overall academic performance. These findings align with earlier studies highlighting reflective thinking as an important factor for academic achievement (Afshar & Farahani, 2015; Chamdani et al., 2022). Notably, students who scored higher on the reflection and critical reflection measures also reported employing more adaptive learning strategies and exhibited increased academic self-efficacy. This is consistent with Kember et al.’s (2000) view of reflective thinking as a method for deriving meaning from educational experiences. Particularly, students who practiced evaluative thinking regarding their performance were found to be more adept at modifying their study habits and addressing academic challenges.

Qualitative data further corroborated these findings, with participants mentioning the use of reflective writing and journaling to evaluate their progress, recognize learning deficiencies, and create time management plans. One student stated, “I began to implement spaced repetition instead of passive reading after considering my exam failures”. This illustrates the relationship between metacognitive awareness and enhanced academic regulation. Furthermore, students who engaged in deeper levels of reflection exhibited greater academic resilience, particularly when faced with unfamiliar academic environments. This supports earlier research by Aydoğmuş and Şentürk (2023), which found that training in reflective thinking improves both responsibility for learning and motivation. In conclusion, reflective thinking contributes to academic success by promoting self-awareness, effective study planning, and strategic learning.

### 5.2. Reflective Thinking in Cultural Adaptation

The results revealed that reflective thinking plays a critical role in how international students navigate cultural transitions, social norms, and academic expectations in China. Both qualitative and quantitative findings support the assertion that reflection enables students to interpret unfamiliar cultural cues and modify their behaviors accordingly. Thematic analysis identified that students used reflective writing to process cross-cultural misunderstandings, develop empathy, and better engage in classroom discourse. For example, one participant shared: “Reflecting on my interactions with Chinese classmates helped me understand their communication style, which was very different from my own”. This aligns with the literature on intercultural competence, which states that reflection facilitates the integration of new cultural knowledge into one’s identity (Lee, 2017). This supports prior intercultural research, which emphasizes that reflection enables students to decode implicit norms and bridge communication gaps (Martin & Nakayama, 2021).

Reflective thinking facilitated students’ emotional and behavioral adaptation. The quantitative data indicate that students scoring higher on the critical reflection subscale reported improved adjustment and comfort within the host culture. This observation aligns with the findings of Guo et al. (2022), who established a significant correlation between reflective awareness and the formation of science identity and epistemological beliefs. Additionally, Orakcı’s (2021) study emphasizes that cognitive flexibility, which is closely associated with reflective thinking, allows learners to alter perspectives and adapt to intricate environments. Evidence of this cognitive adaptability was present in student reflections regarding their adjustment to collectivist classroom norms, various feedback mechanisms, and hierarchical relationships between instructors and students. Overall, the results imply that reflective thinking serves as a connection between academic involvement and cultural immersion, enabling students to effectively navigate new environments with sensitivity, critical awareness, and strategic adjustment.

### 5.3. Reflective Thinking and Language Acquisition

The findings revealed a significant connection between reflective thinking and learning a second language, particularly regarding how international students monitored, adjusted, and enhanced their language skills through self-regulated techniques. This correlation was substantiated by both quantitative data and qualitative observations. Participants who demonstrated higher scores in the reflective and cognitive comprehension aspects of the Reflection Thinking Scale (RTS) also reported increased awareness of their language learning obstacles and the strategies required to overcome them. These individuals noted utilizing reflective journals to document vocabulary growth, identify pronunciation challenges, and evaluate communication breakdowns. One participant remarked: “I started keeping a journal of new words and made an effort to use them in conversations.”

These results are consistent with the research by Li (2023) on the use of metacognitive strategies among Chinese learners, indicating that progress in language learning is bolstered by reflective monitoring and motivation. Additionally, Hammad Al-Rashidi and Aberash (2024) posited that reflective practices enhance learners’ capacity to assess their language proficiency and implement focused improvements in areas such as grammar, pronunciation, and fluency. Furthermore, Chamdani et al. (2022) illustrated that reflective thinking amplifies learners’ self-regulatory awareness, a recognized predictor of second language success. This study supports these previous findings, particularly through the analysis of journal entries where students initiated consistent vocabulary review, sentence restructuring, and pronunciation adjustments. Overall, reflective thinking seems to serve as a cognitive link between experience and language development, facilitating a transition for international students from passive reception to active metacognitive engagement in the language learning process.

### 5.4. Reflective Thinking and Personal Growth

This study’s qualitative findings strongly highlight reflective thinking as a catalyst for international students’ personal development, identity formation, and emotional resilience. Through reflection, students gained a deeper understanding of their values, behaviors, learning habits, and emotional reactions to change. This supports Mezirow’s (1991) theory of transformative learning, which suggests that critical reflection can lead to shifts in perspective and personal empowerment. Several students described journaling as a space for self-dialogue and personal evaluation. For example, one participant remarked: “Each journal entry helped me understand how much I’ve matured since arriving in China”. Such statements reveal that reflective thinking contributed not only to academic adjustment but also to emotional growth and the development of self-efficacy.

This transformation was also evident in how students responded to challenges. Rather than avoiding failure, they used reflection to reframe difficulties as learning opportunities. These practices align with Zimmerman’s (2000) concept of self-regulated learning, in which reflection supports proactive goal-setting, strategy adjustment, and self-monitoring of progress. In addition, emotional regulation was a recurring theme, with students describing reflection as a tool for managing frustration, homesickness, and interpersonal conflicts. Reflective awareness thus enabled them to recognize negative emotions without being overwhelmed, and to take constructive action. Overall, the findings suggest that reflective thinking not only enhances academic outcomes but also fosters psychological resilience, self-confidence, and adaptive identity development traits that are essential for successful international student experiences.

## 6. Conclusions

The objective of this study was to validate the Reflective Thinking Scale (RTS) for international students in China and to investigate how reflective thinking is expressed in academic, cultural, and linguistic contexts. Utilizing a mixed-methods approach, the research confirmed the four-factor structure of the RTS via exploratory and confirmatory factor analyses, demonstrating both internal consistency and temporal stability. Quantitative results showed strong correlations between reflective thinking, academic performance, cultural adaptation, and personal growth. Students demonstrating higher scores in reflection and critical reflection reported enhanced academic self-regulation and resilience. These quantitative findings were complemented by qualitative data that highlighted students’ use of reflection in managing cultural diversity, regulating their learning processes, and addressing emotional challenges.

This research adds to the existing literature that recognizes reflective thinking as a crucial skill for academic achievement and intercultural proficiency. Additionally, this study emphasizes the importance of reflective writing, metacognitive approaches, and structured reflection in creating supportive learning environments for international students. Theoretically, this research expands upon Kember et al.’s (2000) RTS validation within a different cultural framework and supports the principles of Mezirow’s (1991) transformative learning theory and Zimmerman’s (2000) self-regulation model. Furthermore, the introduction of cognitive flexibility as a reflective characteristic enhances the educational framework for promoting learner adaptability in global classrooms.

### Implications for Future Research

The current study offers significant evidence supporting the validity and importance of the RTS in international education; however, there are multiple pathways for additional research. Initially, future studies should aim to replicate the RTS framework in various intercultural environments, specifically in non-Asian and non-university contexts, to assess its applicability across different learner demographics. In addition, employing longitudinal methodologies would enable researchers to monitor the progression of reflective thinking over time, potentially revealing how critical reflection advances through various academic phases. Furthermore, subsequent research might evaluate the impact of scaffolded reflective strategies, including digital portfolios, prompt-based journaling, or guided self-assessment exercises, on improving students’ metacognitive awareness and educational outcomes. Lastly, considering the increasing influence of technology in reflective practices, future investigations could examine the relationship between AI-assisted tools (such as ChatGPT) and the development of reflective thinking, especially in multilingual or virtual learning settings. These research directions would enhance understanding of how to create inclusive, cognitively engaging, and culturally responsive education for global learners.

## Figures and Tables

**Table 1:** Demographic characteristics of participants (*n* = 482).

Demographic Variable	Category	n	Frequency (%)
Gender	Male	254	52.7%
	Female	228	47.3%
Academic Level	Undergraduate	210	43.6%
	Postgraduate	272	56.4%
Age Range	18–20 years	120	24.9%
	21–23 years	280	58.1%
	24 years and above	82	17.0%

**Table 2:** Principal component analysis (PCA).

Component	Initial Eigenvalues			Extraction Sums of Squared Loadings			Rotation Sums of Squared Loadings		
	Total	% of Variance	Cumulative %	Total	% of Variance	Cumulative %	Total	% of Variance	Cumulative %
1	4.829	30.183	30.183	4.829	30.183	30.183	3.235	20.219	20.219
2	2.515	15.720	45.904	2.515	15.720	45.904	2.631	16.445	36.664
3	1.969	12.303	58.207	1.969	12.303	58.207	2.527	15.792	52.456
4	1.491	9.319	67.526	1.491	9.319	67.526	2.411	15.070	67.526
5	0.915	5.716	73.242						
6	0.793	4.958	78.200						
7	0.708	4.428	82.628						
8	0.574	3.587	86.215						
9	0.553	3.457	89.672						
10	0.421	2.629	92.302						
11	0.382	2.386	94.688						
12	0.238	1.486	96.174						
13	0.218	1.364	97.539						
14	0.203	1.267	98.806						
15	0.106	0.666	99.471						
16	0.085	0.529	100.000						

**Table 3:** Factor loadings and communalities of the RTS.

Item #	Factors				Communalities
	Habitual Action	Understanding	Reflection	Critical Reflection	
1	0.877				0.826
13	0.746				0.834
9	0.736				0.501
5	0.584				0.520
2		0.910			0.829
14		0.776			0.614
10		0.638			0.776
15			0.877		0.812
3			0.746		0.817
7			0.736		0.618
11			0.584		0.399
16				0.854	0.810
4				0.765	0.638
8				0.742	0.664
12				0.739	0.574

Note. Loadings < 0.40 are suppressed for clarity. Rotation method: Varimax with Kaiser normalization.

**Table 4:** Correlation matrix.

		Habitual Action	Understanding	Reflection	Critical Reflection
	Habitual Action	-			
	Understanding	0.429	-		
	Reflection	0.905	0.683	-	
	Critical Reflection	0.808	0.689	0.756	-

**Table 5:** Internal consistency and test–retest reliability.

Factor	Cronbach’s Alpha (α)	Test–Retest Reliability (r)
Habitual Action	0.67	0.73
Understanding	0.67	0.80
Reflection	0.77	0.83
Critical Reflection	0.69	0.78
Overall	0.77	0.83

Note. The threshold for acceptable reliability is α ≥ 0.70 (Nunnally, 1994); acceptable test–retest reliability is r ≥ 0.70 (Aaronson et al., 2002; Heale & Twycross, 2015).

**Table 6:** CFA model fit indices for the four-factor RTS model.

Fit Index	Criteria	Measurement Model	Model Fit
CMIN (χ^2^)	-	1181.933	
df	-	291	
χ^2^/df	≤5	4.061	Acceptable
CFI	≥0.90	0.901	Good
GFI	≥0.90	0.900	Good
TLI	≥0.90	0.910	Good
RMSEA	≤0.08	0.073	Acceptable

Note: χ^2^ = chi-square; df = degree of freedom; CFI = Comparative Fit Index; GFI = Goodness-of-Fit Index; TLI = Tucker–Lewis Fit Index; RMSEA = Root-Mean Square Error of Approximation.

**Table 7:** Gender and grade differences in RTS scores.

		N	M	SD	SE	*T*-Test	DF	ρ
**Gender**	Male	254	58.9764	5.65611	0.35490	5.337 ***	480	<0.001
	Female	228	55.8860	7.03767	0.46608			
**Grade**	Under-graduate	210	56.0000	7.99521	0.55172	4.569 ***	480	<0.001
	Post-graduate	272	58.6838	4.81036	0.29167			

*** significance at the level of 0.001.

**Table 8:** Summary of qualitative results.

Theme	Example Quote	Data Source	Description	Interpretation
1. Cultural Adaptation	“I began adjusting how I communicated and participated.”	Interview 4, Journal 8	Reflecting on unfamiliar norms helped improve participation.	Reflective thinking enables students to adapt culturally and integrate more smoothly into new academic environments.
2. Language Monitoring	“I recorded new words weekly and used them in class.”	Journal 3, Journal 10	Journaling supported vocabulary building and self-correction.	Reflective thinking improves language-learning strategies and academic success.
3. Academic Self-Regulation	“I restructured my study plan after reflecting on failures.”	Interview 6, Journal 5	Reflection prompted changes to study routines.	Reflective thinking fosters problem-solving skills and efficient study methods.
4. Scaffolding Needs	“We were told to reflect, but I didn’t know how.”	Interview 7, Journal 12	Students wanted clearer instructions on how to reflect.	Structured support is essential for developing reflective thinking skills.
5. Identity Transformation	“Each journal entry showed me how I’d matured.”	Journal 17, Interview 9	Journaling helped students reevaluate their self-concept.	Reflective journaling helps track personal growth and fosters self-awareness.
6. Emotional Regulation	“Writing helped me adjust how I deal with frustration.”	Interview 11, Journal 15	Students used writing to process stress and peer issues.	Reflective journaling improves problem-solving and self-regulation.

## Data Availability

The datasets used and/or analyzed during the current study are available from the corresponding author upon reasonable request.

## Appendix A. Reflective Thinking Scale (RTS)

The Reflective Thinking Scale (RTS), developed by Kember et al. (2000), is a 16-item self-report questionnaire designed to measure reflective thinking across four dimensions: habitual action, understanding, reflection, and critical reflection. The scale uses a 5-point Likert scale, where respondents rate their agreement with each statement from 1 (strongly disagree) to 5 (strongly agree). Below is the complete RTS:

Instructions: Please indicate the extent to which you agree with each statement by circling the appropriate number (1 = Strongly Disagree, 5 = Strongly Agree).

**Table A1:** Reflective Thinking Scale (RTS).

Item	Statements	1	2	3	4	5
1	I often reflect on my actions to understand how I can improve.					
2	I think about how my actions affect others in group settings.					
3	I reflect on my learning strategies to identify areas for improvement.					
4	I critically analyze my beliefs and assumptions to improve my understanding.					
5	I often think about how I can apply what I’ve learned to real-world problems.					
6	I reflect on my mistakes to avoid repeating them in the future.					
7	I think about how my cultural background influences my learning.					
8	I reflect on my interactions with others to improve my communication skills.					
9	I often question my assumptions to gain a deeper understanding of a topic.					
10	I think about how my learning strategies can be improved.					
11	I reflect on my experiences to gain new insights.					
12	I critically evaluate my actions to ensure they align with my goals.					
13	I think about how my actions contribute to group success.					
14	I reflect on my progress to identify areas where I need to improve.					
15	I often think about how I can apply my knowledge to new situations.					
16	I critically reflect on my beliefs to ensure they are well-founded.					
